# An Overview of the Stability and Fretting Corrosion of Microgrooved Necks in the Taper Junction of Hip Implants

**DOI:** 10.3390/ma15238396

**Published:** 2022-11-25

**Authors:** Mohsen Feyzi, Khosro Fallahnezhad, Mark Taylor, Reza Hashemi

**Affiliations:** College of Science and Engineering, Flinders University, Tonsley, SA 5042, Australia

**Keywords:** microgrooves, stability, fretting corrosion, head–neck junction, hip implant

## Abstract

Fretting corrosion at the head–neck interface of modular hip implants, scientifically termed trunnionosis/taperosis, may cause regional inflammation, metallosis, and adverse local tissue reactions. The severity of such a deleterious process depends on various design parameters. In this review, the influence of surface topography (in some cases, called microgrooves/ridges) on the overall performance of the microgrooved head–neck junctions is investigated. The methodologies together with the assumptions and simplifications, as well as the findings from both the experimental observations (retrieval and in vitro) and the numerical approaches used in previous studies, are presented and discussed. The performance of the microgrooved junctions is compared to those with a smooth surface finish in two main categories: stability and integrity; wear, corrosion, and material loss. Existing contradictions and disagreements among the reported results are reported and discussed in order to present a comprehensive picture of the microgrooved junctions. The current research needs and possible future research directions on the microgrooved junctions are also identified and presented.

## 1. Introduction

Over the past four decades, modularity at the head–neck (trunnion) junction of hip implants has become popular as it enables surgeons to address patient-specific anatomical and geometrical requirements in hip joint arthroplasty [1,2,3]. In addition, modularity enables surgeons to select different materials for the junction components (head and trunnion) [4,5,6] with lower risks/costs in replacement surgeries [7,8]. However, despite these advantages, the modularity is reported to be a root cause of mechanically assisted crevice corrosion (MACC) [9,10,11]. The Morse taper interface of the head–trunnion junction which is highly loaded by physical activities in the presence of a corrosive environment (body fluid) ends up with some metallic ions/debris released from the interface, which consequently causes regional inflammation, metallosis, and adverse local tissue reactions (ALTRs) [4,5,7]. The severity of this damage depends on various factors such as the taper angle mismatch [12,13,14], geometrical dimensions [15,16], surface topography [17,18], the type, direction, and magnitude of the applied loads [19,20], and the assembly force and/or procedure [21,22,23]. The current understanding recommends a well-engaged interlock as one possible solution for minimizing the damage at the junction interface [4,13,14,24].

Surface finish/roughness is one of the key parameters affecting the interface engagement which is of interest because manufacturers traditionally believe that it can improve the junction integrity and durability [4]. There are two main classes of surface finish: (1) trunnions of the same roughness as their head counterparts, and (2) trunnions produced with purposely designed microgrooves (also called ridges/undulations) [25]. The periodic pattern of such microgrooves is sometimes classified with an amplitude and wavelength of more than 4 µm and 100 µm, respectively [25]. These microgrooves were originally created on the trunnion’s surface to minimize the risk of brittle fracture which may naturally occur in ceramic heads. These microgrooves are also believed to subside the stress field in the junction components and increase the engaging area due to the plastic deformation occurring at the tip of the microgrooves, thereby creating possible pointed cold welding [4]. These may subsequently reduce the probability of fluid ingress into the crevice-like gap of the junction due to the toggling effects, thus reducing the susceptibility to corrosion. The presence of such microgrooves is believed to partly compensate for the influence of unavoidable taper angle mismatch which results in a smaller contact area and possible drop in integrity [26,27,28]. Although modern junctions are mostly designed and manufactured with microgrooves, research confirms that there is a limited understanding of how these microgrooves affect the junction performance, as evident in the inconsistent and often contradictory results reported by researchers. Some research studies confirm no significant role played by these microgrooves in the interface damage [29,30] but some others report higher/lower interfacial damage for these junctions [26,27,28]. There is also disagreement on the influence of these microgrooves on the pull/twist-off strength [24,28]. In addition, variations in the design of microgrooves are very evident in the junction batches, even those produced by the same manufacturer [26,27,28].

Taking into account the aforementioned points, the aim of this paper was to integrate the latest research findings in order to give an overview of the influence of microgrooves on the performance of the head–neck junctions. Hence, different approaches taken by various researchers together with their specific limitations and simplifications are presented and compared. The main results and potential reasons for contradictions are discussed in order to provide a more comprehensive picture of the microgrooved junctions, as well as their performance and longevity in actuality. The performance of the junctions is evaluated according to two design metrics categorized into two main subsections: stability and integrity; wear, corrosion, and material loss.

## 2. Taper Junctions of Hip Implants

The taper junction of modular hip implants consists of a femoral head assembled intraoperatively onto a male trunnion [5]. The two main components are usually manufactured from ceramic (alumina/zirconia), and Co28Cr6Mo, 316L stainless steel, and Ti6Al4V alloys [4,5]. There are various types of heads/trunnions on the market with different design characteristics (geometry, material, and surface finish) [25]. In terms of surface finish (which is the focus of this review), the engaged surfaces of the junction components can be machined smoothly or with microgrooves. Normally, the microgrooves are created on the trunnion surface, and the head taper is machined smoothly. Alternatively, the surfaces of both the head and the trunnion components are machined smoothly/with microgrooves. The design characteristics of these periodically created microgrooves (the amplitude and/or the wavelength) together with whether or not both the components are machined smoothly/with microgrooves could positively/negatively affect the lifespan of the taper junctions. Such an influence is reviewed and discussed below.

### 2.1. Stability and Integrity

**Figure 1:**
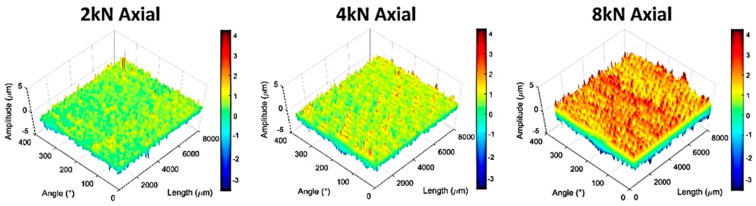
The distortion of microgroove amplitudes on the flattened surface of the trunnion following axial assembly forces with different magnitudes [25].

The stability/integrity of a junction is usually selected to foretell the possibility of postoperative issues in hip arthroplasty. The stability of the taper junctions is compared using various metrics such as the contact situation (contact area and deformation), micromotion, pull-off forces, and twist-off moments [4,5]. There have been few studies with a key focus on investigating the influence of microgrooves on the stability of taper junctions. In some of these studies, this influence was combined with the influence of other variables such as the assembly force, trunnion length, and taper angle mismatch. In a recent study by Dransfield et al. [25], the contact situation was monitored by measuring the deformations of the microgrooves using a method called “roundness measurement”. Impaction forces of 2, 4, and 8 kN were selected to assemble 27 mm CoCr heads onto Ti trunnions. The impaction forces were applied at three different angles: 10° anterior, 10° antero-proximal, and axial. The assembled junctions were then dismantled using an Instron testing machine with an axial tensile load. Of all the test batches, the junction assembled with 8 kN impaction force at 10° antero-proximal had maximal dismantling force. The authors of the study argued that the monitoring of assembly force during the tests was quite challenging; therefore, the dismantling force did not fully represent the pull-off strength. Hence, they selected a ratio of dismantling force divided by the assembly force as a metric for evaluating the junction integrity. Having this ratio determined, they observed the axially assembled junctions as much stronger than those assembled off-axially. At the assembly force of 2 kN, the anterior junctions were stronger than the antero-proximal ones, while, at assembly forces of 4 and 8 kN, the latter was stronger than the former. Global compression of the microgroove amplitudes increased with an increase in the assembly force specifically for the axially assembled junctions (Figure 1). This finding is consistent with the philosophy behind the design and the creation of microgrooved trunnions.

**Figure 2:**
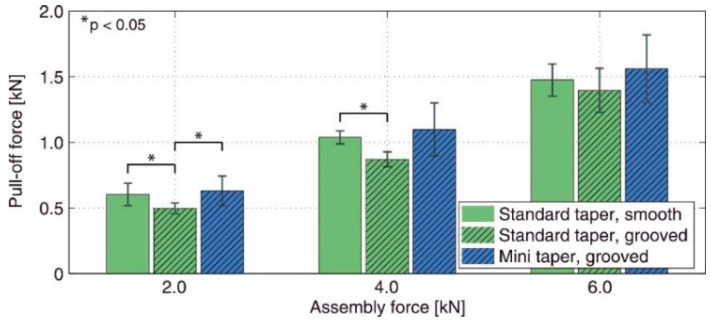
The influence of trunnion length, assembly force, and surface finish on the pull-off strength of 12/14 titanium trunnions assembled onto 28 mm CoCr head counterparts [24].

The coupling between assembly force and microgrooves was also reported by Matt et al. [24]. They evaluated the pull-off strength of a number of customized 12/14 Ti trunnions with different lengths (named standard (14.5 mm) and mini (6.5 mm)) paired with a 28 mm CoCr head. The axial impaction forces were selected in the interoperative range of 2 kN to 6 kN. Three groups of junctions were selected for the experiments: standard/smooth (microgroove amplitude of 7.5 µm), standard/grooved (microgroove amplitude of 15.5 µm), and mini/grooved. The results of this study showed an increase in the pull-off strength with an increase in the assembly force consistent with those reported by Dransfield et al. [25]. When the assembly force was less than 4 kN, the smoothed junctions showed significantly higher strengths compared to the microgrooved ones. At an assembly force of 6 kN, however, no significant change in strength with the surface finish was observed. For comparison, Figure 2 illustrates the difference between the pull-off strength of the junction groups considered in Matt et al. [24]. From this figure, one can see that, when the trunnion length was shorter and the microgrooves were present, the pull-off strength was higher than in other cases, possibly because of the surface microgrooves and the length of the trunnion. A shorter trunnion possibly increases the positive influence of the designed microgrooves, thereby increasing the integrity.

**Figure 3:**
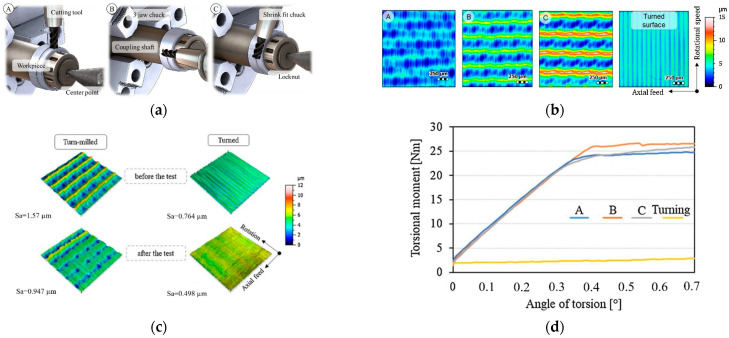
(**a**) Cutting principles for the turned milling process. (**b**) The three surface topographies produced by turned milling process. (**c**) The deformation of surface topographies after the twist-off tests. (**d**) The torque required for dismantling the head–neck junctions [27].

The influence of the microgrooves can also be improved by manipulating the parameters of the microgroove creation procedure. In this regard, the twist-off strength of the turned taper junction was strongly improved by the turn milling method in Döbberthin et al. [27]. It was shown that, as a function of the machining parameters such as the rotational speed and axial feed, the resulted topography on the trunnion surface (and, thus, its integrity) changes significantly (Figure 3a–c). Figure 3d compares the dismantling torques of the junctions with different topographies. From this figure, it can be implied that neither extreme high (“C”) nor extreme low roughness (“A”) resulted in an integrity as the moderate roughness (“B”). This shows the double-sided influence of the created microgrooves on the junction integrity which strongly depends on the design characteristics of the microgrooves.

**Figure 4:**
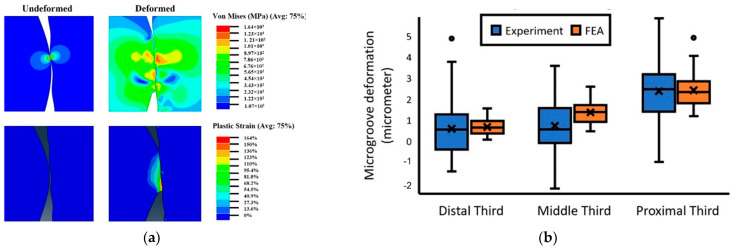
Plastic deformation observed in the microgrooves in Godoy et al. [32]: (**a**) the results of von Mises stress; (**b**) the deformation of the microgrooves in three regions along the trunnion length; circles represent the maximum values (upper extreme) of microgroove deformation.

The published literature shows some studies in which the finite element (FE) approach was used to estimate the contact situation of the microgrooved junctions. Bechstedt et al. [31] observed significant changes in the contact situation of the microgrooved junctions after assemblage. They used the 2D axisymmetric finite element (FE) technique to evaluate the contact situation and the level of micromotions at the interface of a 36 mm CoCr/ceramic head on a Ti trunnion assembled with various forces of 0.5, 2, 4, and 8 kN. The microgrooves of the trunnion were modeled using sinusoidal periodic waves with an amplitude of 11 µm and a spacing of 200 µm. For the head tapers, the amplitude and spacing were considered as 10 µm and 220 µm, respectively. The model was validated against experimental data for the contact situation. Once verified, the results for the three surface topographies with heights of 2, 1, and 14 µm showed the pivotal role played by the assembly load in altering the contact area, whereas the deeper microgrooves resulted in a smaller contact area (partly against the main philosophy behind the microgrooves). For the junctions with a CoCr head, all microgrooves were in contact even with the lowest assembly force; however, for the cases with a ceramic head, few microgrooves were in contact. This was mainly due to the deformation of both components and their possible interactions in the junctions with a metallic head. Overall, only few numbers of the microgrooves showed plastic deformation, and the level of plasticity increased with an increase in either the assembly force or the microgroove heights. Godoy et al. [32] used a 2D axisymmetric model with a sinusoidal pattern of microgrooves on a trunnion with an amplitude and period of 33 µm and 310 µm, respectively. The model included a roughness of 0.33 µm on the surface of the head tapers. They verified the FE results against experimental data from interferometry for 28 mm CoCr heads assembled onto 12/14 Ti trunnions with an impaction force of 6 kN. The results of microgroove deformations indicated a good agreement between the FE predictions and experiments such that the mean changes in the microgroove heights from FE and measurements were 1.40 µm and 1.23 µm, respectively. Furthermore, 76–89% and 91–100% of all the microgrooves were deformed according to the experimental and FE results, respectively, which is partly inconsistent with the findings in [31]. The study conducted by Godoy et al. [32] clearly showed the importance of the microgroove heights on the global deformations as reported in [31]. Plastic deformation was noted in the FE models at the tip of the microgrooves, as illustrated in Figure 4a. Figure 4b shows the degree of deformation in the microgroove in the distal third, middle third, and proximal third regions. Higher deformation in the proximal third was due to the positive mismatch angle between the trunnion and head taper. This shows a possible interaction between the design of the microgrooves and the taper angle mismatch. In addition to the taper angle mismatch, the deformation and contact pressure were indicated to be a function of the magnitude of assembly force in Gustafson et al. [33]. The change in assembly force from 4 kN to 12 kN changed the contact pressure (from 803.9 MPa to 964.8 MPa) and plastic strains (from 0.6% to 6%). However, the change in the number of hits from one to three did not significantly alter these parameters. The model in [33] was recently used in Gustafson et al. [34] to evaluate the influence of taper angle mismatch and microgroove pattern on the junction integrity. For the trunnion, four microgroove patterns were selected as follows: (amplitude, spacing) = (2, 30), (6, 150), (11, 200), and (14, 200) µm. The head taper was modeled as either “ideal/flat” or with an amplitude and spacing of 2 µm and 50 µm, respectively. Taper angle mismatches of −0.2°, −0.05°, 0°, +0.05°, and +0.2° were considered for the models. When comparing the contact area, the influence of trunnion microgroove pattern was the most important factor followed by the presence of microgrooves on the head taper. The taper angle mismatch did not show a significant effect on the contact area; however, its influence was observed in the maximal contact pressure. The presence of taper microgrooves and the taper angle mismatch played key roles in the plastic strain magnitudes and distributions, which is consistent with the findings in Godoy et al. [32].

**Figure 5:**
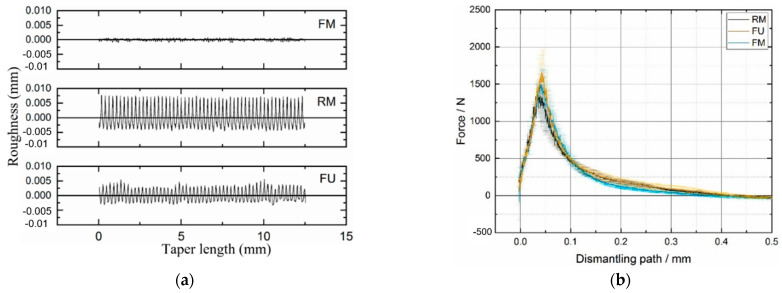
(**a**) Three surface topographies machined on the 12/14 Ti trunnions. (**b**) The dismantling force for the three types of the assembled junctions (Mai et al. [28]).

Both the FE and the experimental results obtained in the aforementioned studies clearly emphasized the influence of various design parameters such as the assembly force, taper angle mismatch, and material couple on the level of effectiveness of the microgrooves in enhancing the junction integrity. In contrlast, there are some studies which highlighted the negative or neutral influence of the microgrooves on the junction integrity. Mueller et al. [35] conducted an extensive investigation on the influence of contact situation (proximal, distal, and full contacts), trunnion topography, head material (ceramic/metallic), and impaction force on the stability of the junctions. The stability was measured using a twist-off testing approach. Four surface topographies (smooth, symmetric rough, asymmetric rough, and very rough) were considered for the 12/14 Ti trunnion which were then coupled with 32 mm ceramic and CoCr heads in proximal contact with assembly forces of 1, 3, and 6 kN. They reported the level of assembly force as the most important parameter in determining the twist-off strength followed by the head material. For higher assembly forces and in the case of having CoCr heads, higher twist-off strengths were observed, which partly support the findings in [31]. Interestingly, no significant influence of the surface topography on the twist-off moment was reported. This result was also partly confirmed by Mai et al. [28], who found that the surface topography did not significantly change the stability of CoCr–Ti head–neck junctions (assembled with 3 kN). Three surface topographies named fine machined (FM), rough machined (RM), and furrowing (FU) (Figure 5a) were created on the surface of the 12/14 trunnions. Figure 5b shows the maximal (1754 N) and minimal (1465 N) dismantling forces for the furrowing and rough machined taper junctions, respectively. This figure partly shows the double-sided influence of the microgroove pattern as observed and reported in Döbberthin et al. [27] with somewhat similar assembly forces in [27] (4 kN) to those used in Mai et al. [28]. In a study by Falkenberg et al. [36], it was demonstrated that the presence of microgrooves (amplitude of 30 µm) did not produce any significant changes in the micromotions at the head–trunnion interface under various taper angle mismatches of 0.052°, 0.100°, and 0.134° and assembly forces of 2, 4, and 6 kN. Although the microgroove heights, taper angle mismatch, and the assembly force used in [36] are well comparable with those in [32,34], no significant contributions from the microgrooves were observed.

The results reported in the aforementioned studies indicated that the microgrooved junctions have either a positive or a neutral effect on the junction integrity. Changing the influence from neutral to positive is influenced by various design parameters. The experimentation of all the possible design shapes is not feasible in reality. As the FE approach has shown its capability in predicting the overall deformations of the microgrooves, it would be wise if these models are more developed to see what happens if the design variable would change in the practical regions using stochastic FE analyses similar to the study carried out for the smoothed junctions by Donaldson et al. [37]. It also seems that the roughness of the surface should not be low or high, and a moderate level is preferred; however, this also depends on the machining method in creating the microgrooves as in the studies of Mai et al. [28] and Döbberthin et al. [27], where the amplitude of around 7 µm resulted in different behaviors of the junction. The reason for the inconsistent findings of these studies could be that the microgrooves created on the head taper surface might change the interface behavior and subsequent effects. Some of these studies considered the roughness of the head tapers, whereas others did not account for this parameter. This requires more attention in experimental/numerical studies. Furthermore, some studies have investigated the influence of microgrooves on only the permanent deformations of the microgrooves, whereas, for a better comparison, one needs to compare the pull/twist-off strengths of the junctions. This is because the plastic deformations are not direct metrics for evaluating the junction integrity.

### 2.2. Wear, Corrosion, and Material Loss

**Figure 6:**
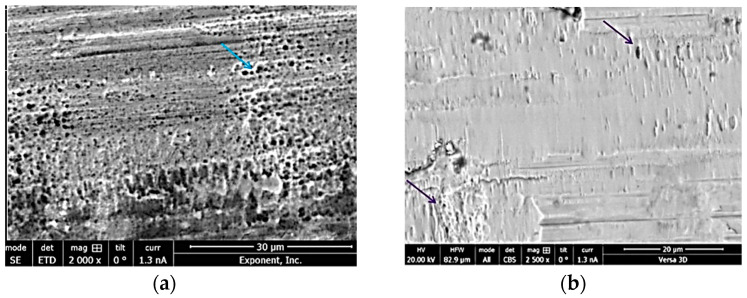
(**a**) SEM image of the fretting damage (blue arrow) on the head surface in the valleys of microgrooved junctions. (**b**) SEM analysis confirms the smeared fretting damage (purple arrows) on the head surface in the smooth junctions [30].

**Figure 7:**
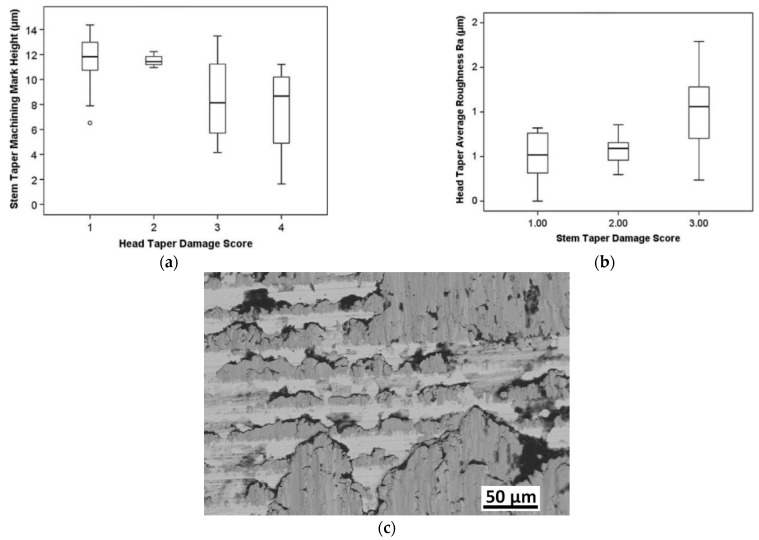
Influence of surface roughness on the damage score observed and measured in Pourzal et al. [39]: (**a**) CoCr–CoCr and (**b**) CoCr–Ti. (**c**) Backscatter mode of SEM on CoCr surface in a CoCr–Ti junction; the CoCr surface (bright areas) includes some Ti (dark areas) transferred from the trunnion.

**Figure 8:**
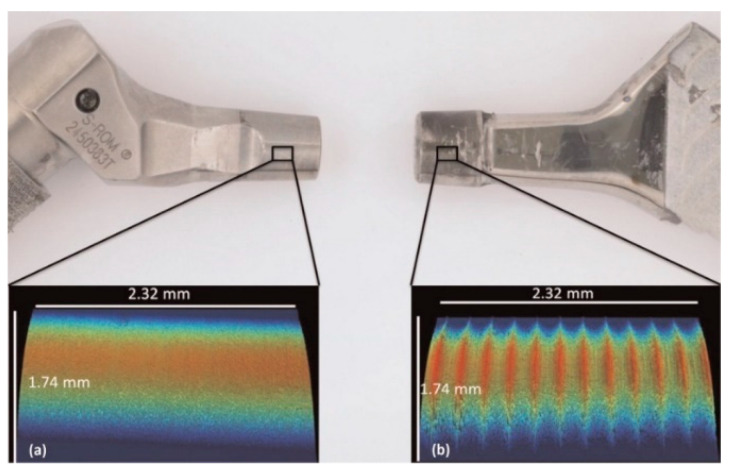
A comparison between the surface topography of the two trunnions considered for visual study in Hothi et al. [41]: (a) smooth, and (b) rough corail tapers.

The stability and integrity of taper junctions are evaluated by indicative metrics mainly in order to predict the durability of the junctions against the wear/corrosion damage mechanism occurring at the interface. Although useful and indicative, these metrics do not provide a comprehensive indication of the junction performance. The wear/corrosion phenomenon is a synergistic degradation process through which the mechanical abrasion, electrochemical repassivation/dissolution, and mechanical–electrochemical interrelations contribute to the total material loss at the interface. Therefore, in this section, the studies on the wear/corrosion of microgrooved junctions are reviewed, and their main results are presented. In this regard, an extensive cohort study was conducted by Arnholt et al. [30] through which 120 junctions were scored according to the Higgs–Goldberg method for determining fretting corrosion damage. The junctions were classified into two main groups, each containing 60 junctions. In one group, the mating trunnion was smooth (with an amplitude and wavelength less than 4 µm and 100 µm), while, in the other, the microgrooved trunnions (with an amplitude and wavelength more than 4 µm and 100 µm) were used. The trunnions and heads were made up of Ti/CoCr and CoCr alloys, respectively. The observation showed no significant difference in the maximum depth of material removal and fretting corrosion damage score between the two groups (for both the female and male tapers). The signs of damage were, however, more visible on the microgrooved tapers. Both groups showed signs of micromotions, fretting corrosion damage, and localized chromium-rich oxide layers, which were not influenced by the surface topography of the trunnions (Figure 6). Conversely, Panagiotidou et al. [38] reported the surface topography as an important parameter affecting the wear/corrosion behavior of the CoCr–Ti head–trunnion interfaces. The CoCr heads were of 28 mm in diameter and mated with 12/14 Ti trunnions (rough/standard) for in vitro tests, through which a sinusoidal load oscillating between 0.1 kN and 3.1 kN was applied to the junction (immersed into PBS solution) for 10 million cycles at the frequency of 4 Hz. The *R_a_* roughness of head taper and rough trunnion were reported as 0.58 µm and 2.73–2.79 µm, respectively. After the in vitro tests, the surface roughness of the head tapers significantly increased where a rough trunnion was used. For the corrosion tests, two in vitro tested junctions were then loaded by a sinusoidal regime fluctuating between 0.1 and 1.5 kN with a frequency of 0.66 Hz for 1000 cycles. The corrosion tests included the open-circuit potential (OCP), potentiostatic tests at 200 mV, and a pitting scan. The results of these tests showed the fracture of the oxide layer (and, consequently, electrochemical repassivation) where a rough trunnion was used. Overall, the use of the rough trunnion exacerbated the crevice environment, resulting in more electrochemical reactions (drops in OCP, creation of potentiostatic current, and hysteresis loop in pitting scan). Therefore, the material loss in junctions with rough trunnions could possibly originate from the mechanical wear, corrosion, and their interrelations, whereas, in the junctions with standard trunnion, the role of mechanical wear seems to predominate the role of corrosion. This is somewhat consistent with the results found by Brock et al. [18], where rough trunnions represented higher volume loss rates (0.402 mm^3^ vs. 0.123 mm^3^ per 1 year). The diameter of the CoCr heads of this study [18] was between 36 mm and 63 mm, mated with either 11/13 or 12/14 Ti trunnions. Overall, the fretting corrosion damage in head tapers was higher than that in the trunnions [18]. Considering the role of material couple, Pourzal et al. [39] more extensively investigated the wear/corrosion damage in 269 head tapers and trunnions classified into CoCr–CoCr and CoCr–Ti head–trunnion junctions. Head diameters were between 28 mm and 50 mm, and the trunnions were of 12/14 and 14/16 proximal/distal diameters. Their results turned out interesting patterns for the damage in both material combinations. In CoCr–CoCr junctions, rougher trunnions resulted in lower damage scores (resulting from wear and corrosion) in head tapers compared to the smooth ones. For the CoCr–Ti junction, rougher head surfaces were associated with higher damage scores in both the head and the trunnion components, whereas increasing the roughness of the trunnion entailed lower damage scores in trunnion. Overall, it was observed that the damage scores of CoCr–CoCr junctions were higher compared to those of CoCr–Ti junctions. More distinct damage observed in CoCr/CoCr couples was related to the higher susceptibility of CoCr to different corrosion mechanisms. Higher fretting corrosion damage in CoCr has also been reported by Kop et al. [40] in both smooth and microgrooved devices; however, they raised the cold welding in the case of using Ti devices. The influence of the surface roughness for the two material combinations obtained in Pourzal et al.’s study [40] is illustrated in Figure 7. Form this figure and according to the results of Kop et al. [40], material combination is a key factor in determining the influence of the microgrooves on the damage severity at the interface. Figure 7c confirms the material transfer from the Ti surface to CoCr surface (the influence of material combination), which can then change the influence of the microgrooves on the junction performance. The contribution of roughness to higher material losses at the metal-on-metal junctions has been also reported by Hothi et al. [41] where they related the higher volume losses in Corail to their rougher and shorter trunnions (height and spacing of ~11.5 µm and 0.2 mm) in comparison with those in S-ROM (height and spacing of ~1 µm and 0.099 mm). However, the shorter trunnion was observed to offer a better integrity in Matt et al. [24]. Therefore, these two observations might be more related to the microgrooves. Figure 8 shows a general comparison of the surface roughness of the two groups considered by Hothi et al. [41].

**Figure 9:**
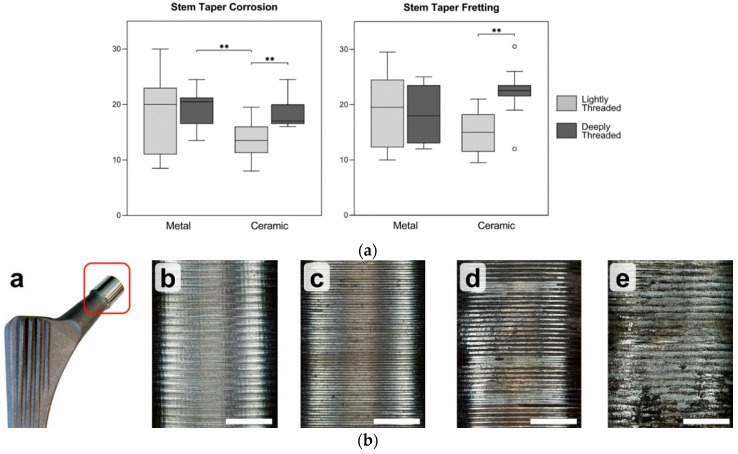
(**a**) The influence of the surface roughness on the fretting and corrosion damage of the trunnions mated with ceramic and metallic heads in Stockhausen et al. [26]. Double asterisks and circles denote highly significant differences (*p* ≤ 0.01), and maximum/minimum damage, respectively (**b**) Four main categories for classification of the damage severity: no damage, mild, moderate, and severe, respectively, from left to right.

In addition to Hothi et al.’s study [41], the variations of the surface topography were also raised in [26]. In a retrieval study, Stockhausen et al. [26] reported considerable variations in the surface topographies of different designs for the taper junctions. This research study was conducted on 46 Ti trunnions with a 12/14 design. They studied the influence of stem topography mated with ceramic/metal heads on the severity of fretting corrosion damage. It was observed that stems mated with ceramic heads were less damaged (in the form of both fretting and corrosion) if they were coupled with a smoother trunnion, while, in the case of having a metallic head, there was no meaningful influence of the surface roughness on the intensity of the fretting and corrosion damage scores (Figure 9a). The scoring method was based on the approach proposed by Goldberg et al. [42] through which the damage intensity was classified into four main categories: no damage, mild, moderate, and severe, as illustrated in Figure 9b. The fretting and corrosion damage scores were determined using Cohen’s kappa tests.

**Figure 10:**
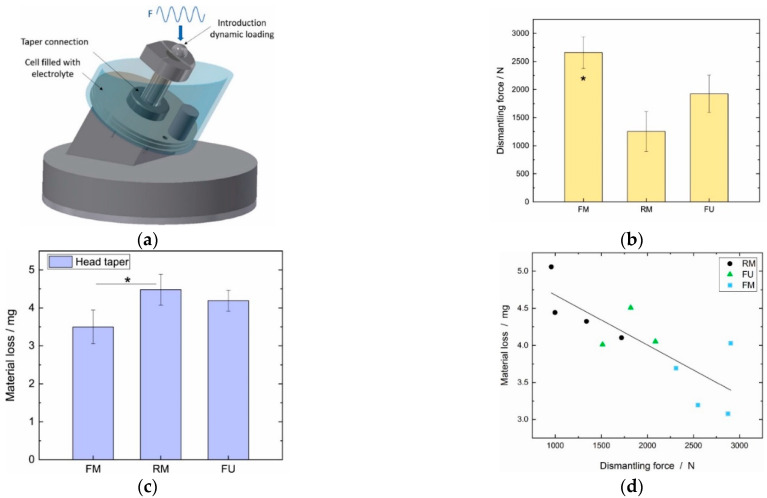
(**a**) Schematic representation of the fretting corrosion tests conducted by Mai et al. [28]. (**b**) The dismantling force after completing the cyclic tests for the three surface topographies. (**c**) The material loss at head taper for the three surface topographies. The asterisk denotes statistically significant differences (*p* ≤ 0.05) (**d**) The correlation between the material loss at the head taper and the dismantling force after the cyclic tests.

In the recent study by Mai et al. [28] (detailed in Section 2.1), a series of in vitro fretting corrosion experiments were conducted to elucidate the influence of surface topography on the severity of the damage. As shown schematically in Figure 10a, an off-axial sinusoidal load oscillating between 300 N and 2500 N with a frequency of 4 Hz was applied to the junction immersed into an acidic solution enriched with chloride ions (pH of 2.9) for 5 million cycles. After completing the fretting corrosion tests, the junctions were dismantled. It was observed that the stability was maximal for the fine machined junctions (2660 ± 284 N) (named “consolidated junctions”), followed by furrowed ones (1925 ± 334 N), followed by rough machined ones (1253 ± 355 N) (Figure 10b). Consistent with the previous studies, the material loss increased with an increase in the surface roughness such that the maximal material loss occurred for the rough machined junctions followed by furrowed and fine machined samples (Figure 10c). Higher material losses in junctions with rough trunnions were related to the higher possibility of solution ingress into the interface. Metal-on-metal junctions were suggested to be used with smoother trunnions because the metal-on-metal junctions are more susceptible to corrosion as confirmed in Pourzal et al. [39]. Interestingly, a correlation was found between the dismantling force after the fretting corrosion tests and the material losses at the interface (Figure 10d). Hence, a higher dismantling force results in less material loss. Comparing the results in Figure 5b and Figure 10b, it can be seen that the influence of the surface topography on the dismantling force changed upon applying the cyclic tests.

Higher wear rates in the microgrooved junctions in comparison with the smoothed junctions were also observed in one FE study by Ashkanfar et al. [43]. In their study, a CoCr–Ti head–trunnion junction was assembled by 4 kN impaction force, and a distal contact with a mismatch angle of −0.05° was considered. The head was 36 mm in diameter, and it was mated with a 12/14 trunnion. Under the walking loads, the fixation of the microgrooved junction was lost after a number of cycles; therefore, the micromotions at the interface escalated. The presence of ridges and their influence on the wear depth were also modeled by Zhang et al. [44] using a sub-modeling technique. It was shown that the wear depth in the sub-model was higher compared to its corresponding value in the global head–neck junction model. A recent FE study by Capitanu et al. [45] also confirmed higher wear rates for the microgrooved junctions consistent with those in [43,44]. In all FE simulations [43,44,45], the role of corrosion was neglected, and the total loss was assumed to originate from the mechanical wear only. Fretting corrosion damage, as a synergistic process, is believed to be significantly affected by the electrochemical corrosion, and this needs to be somehow included in future FE simulations to produce a better picture of the influence of microgrooves.

The comparison of all studies above seems to signify a common message of higher volume losses for microgrooved junctions. In Section 2.1, it was observed that the stability and integrity of the junction are positively influenced by the microgrooves, while, in this section, the fretting corrosion of such junctions was more severe (except some research cases [29]). It should be noted that the studies reviewed in this section were mostly visual-based (except the FE and in vitro ones) through which the design parameters (such as the head size, trunnion flexural rigidity, and material combinations) were largely different, whereas, in Section 2.1, it was concluded that a small change in each of the design parameters changes the whole mechanical behavior of the junction. Furthermore, in some of the studies in this section, the loading history of the inspected junctions was not clear, and this may have again changed the overall conclusions. More specific studies are required to conduct one-to-one comparisons between the microgrooved and smoothed junctions to derive a more valid final conclusion. The FE study conducted by Ashkanfar et al. [43] showed higher fretting corrosion damage in microgrooved junctions; however, this needs more research as the design parameters are very specific and limited, while the variations among the designs are very large and well documented [26,27,28,46].

## 3. Discussion

Surface topography is one of the key design parameters which significantly affects the performance of head–neck junctions. The surface topography is sometimes designed to purposefully enhance the junction integrity and its longevity [4,5,30]. These are commonly called microgrooved/ridged junctions. The mechanical performance of the microgrooved junctions versus smoothed junctions was recently raised as a research question [27,30,31,36,43]. Previous findings and reported results are contradictory; furthermore, there is no agreement on the microgroove geometry. This study was conducted to provide an overview of the latest findings of the microgrooved junctions, and it categorized the research studies according to two main metrics: stability and integrity; wear, corrosion, and material loss. According to this overview, some research studies support the main philosophy behind the creation of the microgrooves to enhance the junction integrity [24,25,27,31] while others report that microgrooved junctions reduce the integrity [28,36]. It seems that, using experimental and/or numerical approaches, most of the reviewed studies concluded that microgrooves have a positive effect on the integrity. However, this positive influence seems to strongly depend on other design parameters such as the taper angle mismatch [34], assembly force [24,25], trunnion geometry [18], and head size/material [31,36]. The interactive influence of these parameters was also noticed such that the influence of the microgrooves was significant in some cases and insignificant in others. This clearly shows a need for further research to provide more extensive analyses to find out the interaction of these parameters. The FE method has shown its capability in predicting the behavior of the microgrooved junctions [4,5,31,33,34,36]; hence, it can be used as a useful tool to explore the change in the design parameters and find out the possible interactions leading to a final change in the junction performance. This modeling procedure might be concluded with an optimal pattern for the microgroove geometry depending on the operational and geometrical constraints together with the material combination of the problem in hand. However, the FE models are time-consuming to complete specifically, where a 3D model is supposed to be used with the inclusion of other geometrical imperfections such as the degree of non-roundness and surface waviness. Furthermore, most of the models are limited to the taper junctions where the surface topography of the head taper is neglected, while this parameter can change the overall conclusions, as observed in previous research [31,47]. The FE models of microgrooved junctions are still in their infancy, and they do not accurately reflect what occurs in reality. In operation, the junction is typically assembled off-axially with head tapers for which the roughness is not negligible. Then, the junction undergoes cyclic loads including both the frictional forces and the moments of the physical activities [4,5,19,20] in the corrosive body medium. Some of these activities, together with higher body weights, might result in critical stress and strain fields, which may then change the influence of microgrooves on the integrity of the junction [4,5]. Furthermore, due to the cyclic action of the loads from physical activities, the junction needs to be analyzed progressively, and the process of interfacial damage needs to be encountered by the future FE models. The FE work completed by Ashkanfar et al. [43] addresses the mechanical wear at the interface of a microgrooved junctions; however, it does not include the head taper roughness and is limited to a geometrical and loading condition, such as that recently conducted by Capitanu et al. [45]. This study [45] also neglected the inclusion of head taper roughness into the modeling phase and was limited to the modeling geometry and material combination. The chapter of the microgrooved junctions is still open, and more research needs to be conducted in similar wear algorithms with possible inclusion of the electrochemical reactions at the interface. The inclusion of the electrochemical reactions at the interface was recently applied to a smoothed CoCr/CoCr head–neck junction by the authors [11]. In this algorithm, the mechanical and electrochemical wear equations were combined into a unique algorithm. It was concluded that the electrochemical reactions are responsible for almost 32% of the total material loss at the interface, and this percentage changes with various design parameters. The basic data for such a modeling procedure can be obtained by fundamental tribocorrosion studies in the ball-on-disc configuration. The role of mechanical and electrochemical reactions in the total tribocorrosion loss changes with various parameters such as the imposed potential [48,49], normal force (and, thus, contact pressure) [50,51], sliding distance and its frequency [52,53,54], material couple in contact [55,56,57,58], and the solution type and its acidity [59,60,61]. However, the design parameters of head–neck junctions including taper angle mismatch, head size, trunnion geometry, material couples in contact, and the solution acidity, together with the presence of proteins, could affect the tribological characteristics of the system, the governing potential, and the degree of mechanical and electrochemical damage processes. These complexities need to be comprehensively included in the experimental tests before the incorporation of the experimental data into the numerical models. In the presence of the microgrooves, the role of the mechanical and electrochemical reactions in the total material damage at the interface might be increased and/or decreased. This, together with the influence of the microgrooves on the gap opening (allowing body fluid ingress into the crevice-like geometry of the junction), needs to be addressed in future modeling studies. This modeling procedure might then generate a more conclusive comparison between microgrooved and smoothed junctions. As evidenced by the FE approach, the interaction of the parameters plays a pivotal role in determining the positive/neutral/negative influence of the microgrooves. Considering the retrieval studies, they were mostly associated microgrooved junctions (with various design parameters) with higher damage intensities. Although being indicative and useful, most of the retrieval studies conducted on the microgrooved junctions focused on a class of junctions with various geometrical parameters (e.g., head size and trunnion geometry), and they sometimes did not give details on the geometry of the microgrooves and/or the loading history of the junction. Keeping the strong interactions of the design parameters in mind, the microgrooved junctions need to be studied more meticulously with possible inclusions of the complexities in both the operational and the post-operational phases. More in vitro studies also need to be conducted in order to provide possible validations for the tribocorrosion-based FE algorithms in simplified oscillatory loading conditions. The validated FE models can then be reliably sophisticated with other parameters to predict the influence of different microgroove designs on the junction longevity and durability in reality.
